# Autophagy Regulates Proteasome Inhibitor-Induced Pigmentation in Human Embryonic Stem Cell-Derived Retinal Pigment Epithelial Cells

**DOI:** 10.3390/ijms18051089

**Published:** 2017-05-19

**Authors:** Kati Juuti-Uusitalo, Ali Koskela, Niko Kivinen, Johanna Viiri, Juha M. T. Hyttinen, Mika Reinisalo, Arto Koistinen, Hannu Uusitalo, Debasish Sinha, Heli Skottman, Kai Kaarniranta

**Affiliations:** 1 Faculty of Medicine and Life Sciences, BioMediTech, University of Tampere, 33014 Tampere, Finland; kati.juuti-uusitalo@staff.uta.fi (K.J.-U.); heli.skottman@staff.uta.fi (H.S.); 2 Department of Ophthalmology, Institute of Clinical Medicine, University of Eastern Finland, 70211 Kuopio, Finland; ali.koskela@uef.fi (A.K.); niko.kivinen@uef.fi (N.K.); johanna.viiri@uef.fi (J.V.); juha.hyttinen@uef.fi (J.M.T.H.); 3 School of Pharmacy, University of Eastern Finland, 70211 Kuopio, Finland; mika.reinisalo@uef.fi; 4 SIBS Labs, University of Eastern Finland, 70211 Kuopio, Finland; arto.koistinen@uef.fi; 5 Department of Ophthalmology, University of Tampere, SILK, TAUH Eye Center, Tampere University Hospital, 33014 Tampere, Finland; hannu.uusitalo@uta.fi; 6 The Wilmer Eye Institute, The Johns Hopkins University School of Medicine, Baltimore, MD 21287, USA; debasish@jhmi.edu; 7 Department of Ophthalmology, Kuopio University Hospital, 70029 Kuopio, Finland

**Keywords:** autophagy, macula, melanosome, proteasome, stem cell

## Abstract

The impairment of autophagic and proteasomal cleansing together with changes in pigmentation has been documented in retinal pigment epithelial (RPE) cell degeneration. However, the function and co-operation of these mechanisms in melanosome-containing RPE cells is still unclear. We show that inhibition of proteasomal degradation with MG-132 or autophagy with bafilomycin A1 increased the accumulation of premelanosomes and autophagic structures in human embryonic stem cell (hESC)-derived RPE cells. Consequently, upregulation of the autophagy marker p62 (also known as sequestosome-1, SQSTM1) was confirmed in Western blot and perinuclear staining. Interestingly, cells treated with the adenosine monophosphatedependent protein kinase activator, AICAR (5-Aminoimidazole-4-carboxamide ribonucleotide), decreased the proteasome inhibitor-induced accumulation of premelanosomes, increased the amount of autophagosomes and eradicated the protein expression of p62 and LC3 (microtubule-associated protein 1A/1B-light chain 3). These results revealed that autophagic machinery is functional in hESC-RPE cells and may regulate cellular pigmentation with proteasomes.

## 1. Introduction

The retinal pigment epithelial (RPE) cells, which are situated between the photoreceptor cells and choroid in the back of the eye, are vitally important for vision by maintaining the viability of photoreceptor cells [1]. Central characteristics of RPE cell degeneration associated with aging include subtle changes in pigmentation, as well as accumulation of intracellular lipofuscin, a highly cross-linked aggregate of oxidized proteins, and extracellular drusen deposits. Accumulation of toxic lipofuscin and drusen in aged RPE cells is suggested to occur as a result of disturbed proteasomal or lysosomal degradation systems, including autophagy [2,3,4,5]. 

Autophagy is essential for maintaining healthy cell homeostasis: the autophagic process begins with the formation of isolation membranes known as phagophores [6], which then become elongated and form mature double membrane autophagosomes. These autophagosomes engulf portions of cytoplasm containing oligomeric protein complexes and organelles, fuse with lysosomes, and their content is then degraded by lysosomal enzymes. Daily phagocytosis of engulfed photoreceptor outer segments (POS) and their degradation in lysosomes (heterophagy) by RPE are critical for maintaining visual functions [7,8]. However, the POS-derived A2E (*N*-retinylidene-*N*-retinylethanolamine) and oxidatively-damaged proteins accumulate and impair acidification of the lysosome, weakening its degradation capacity [9,10,11] and lysosomal enzyme activity [12,13]. Failure of autophagy in aged post-mitotic cells, including RPE cells, can result in accumulation of lipofuscin, which in turn can further diminish lysosomal enzyme activity [9,14] and lead to cellular degeneration and finally to cell death [2,5]. A decline in autophagy is usually accompanied by the accumulation of aggregates expressing autophagy markers p62 (also known as sequestosome-1, SQSTM1) and LC3 (microtubule-associated protein 1A/1B-light chain 3, also known as MAP1LC3A), normally degraded by lysosomal enzymes in autophagic processes [3,5,15]. 

RPE cell pigmentation mainly results from melanosomal melanin [16], which acts as a scavenger of reactive oxygen species (ROS) and chelator of metals, thereby protecting neural retina from oxidative damage [16,17,18]. During aging, as a result of increased oxidative stress and decreased melanin content, the antioxidant capacity is estimated to decline in the retina [19,20,21]. Moreover, increased melanosomal oxygen consumption and ROS production have been observed in aged RPE cells. This in turn is estimated to occur due to melanosomal lipofuscin accumulation onto the surface of melanosomes in RPE cells [22,23,24]. Previously, it was believed that lipofuscin aggregates could not be degraded by proteasomal or lysosomal enzymes, nor transported into the extracellular space via exocytosis [25]. However, there are indications that RPE cell melanolipofuscin might be exocytozed and degraded by macrophages or complexed with β-cyclodextrins and removed [26,27]. Furthermore, recent findings have revealed that both proteasomes and autophagy takes part in the regulation of cellular pigmentation [28,29,30,31].

RPE-like cells, expressing genes and proteins corresponding to the human RPE [32,33,34], have been successfully differentiated from human embryonic stem cells (hESCs) and human-induced pluripotent stem cells (hiPSCs) [32,35,36]. In addition, highly pigmented hESC-RPE and hiPSC-RPE cells exhibit functions that are characteristic to native RPE cells: they are able to phagocytose POS, secrete RPE trophic factors such as pigment epithelium-derived factor and form a tight epithelium with high electrical resistance [32,33,34,37,38,39,40]. However, the functionality of autophagic and proteolytic machinery in the regulation of melanocytic pigmentation in hESC-RPE cells is yet to be determined. In this study, co-operation of proteasome and autophagy machinery in the regulation of melanocytic pigmentation in mature hESC-RPE cells was assessed.

## 2. Results

### 2.1. Human Embryonic Stem Cells-Derived Retinal Pigment Epithelial Cells Show the Typical Retinal Pigment Epithelial Phenotype and Express Retinal Pigment Epithelial Specific Genes

**Figure 1:**
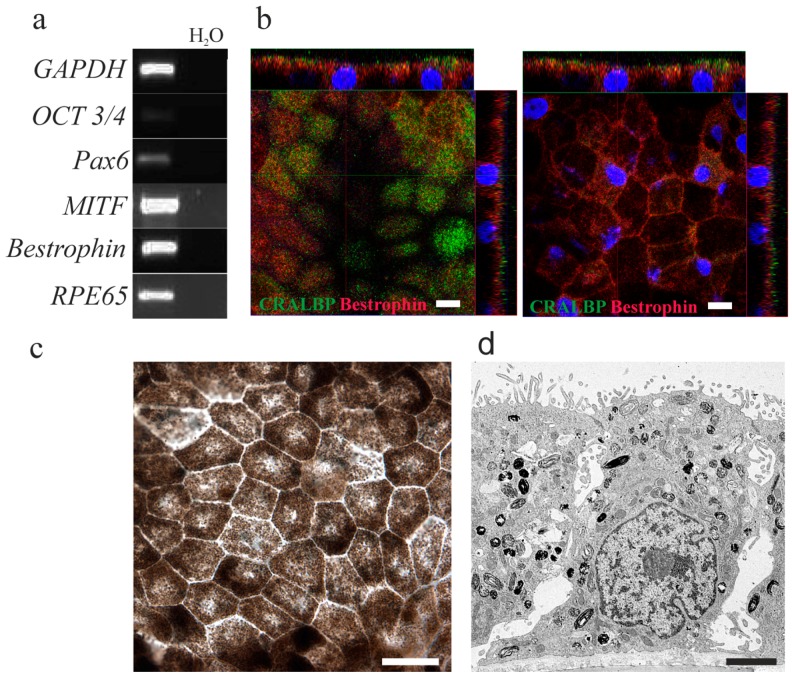
Representative brightfield micrographs and gene expression of human embryonic stem cells (hESC)-derived retinal pigment epithelial (RPE) cells used in this study. (**a**) Reverse Transcription-Polymerase Chain Reaction gene expression analyses of mature hESC-RPE cells used for the analyses (analyzed genes on the left, water control on the right); (**b**) confocal micrographs after indirect immunofluorescence with RPE-specific proteins cellular retinaldehyde-binding protein (CRALBP, green) and bestrophin (red), and the nuclear label DAPI (blue, 4′,6-diamidino-2-phenylindole). Scale bar = 10 µm. On the left-hand side is the image from basal side of hESC-RPE cells with blue stained nuclei, and on the right-hand-side is the same sample from the apical side of cells. (**c**) A brightfield phase contrast micrograph of representative cell morphology. (**d**) A low resolution electron microscopy image of hESC-RPE cells cultured on inserts, showing a high degree of polarization with apical brush border and apico-lateral tight junctions, structured organization of basal extracellular matrix and a high number of pigmented granules. Scale bar = 2 µm.

In this study, our aim was to assess the functionality of mature hESC-RPE cells; therefore, it was important to verify that the used hESC-RPE cells have been successfully differentiated and matured from pluripotent stem cells. This was done by assessing gene expression with Reverse Transcription-Polymerase Chain Reaction (RT-PCR), protein expression and localization with immunofluorescence labelling, confocal microscopy and evaluating cell morphology and pigmentation with brightfield phase contrast microscopy and Transmission Electron Microscopy (TEM). Reverse Transcription-Polymerase Chain Reaction gene expression analysis revealed that hESC-RPE cells did not express the *Oct3/4* marker of pluripotency and only a minute amount of eye specific lineage marker *PAX6*. All of the analyzed RPE-specific genes, namely *MITF*, *Bestrophin* and *RPE65*, were expressed by hESC-RPE cells (Figure 1a). The gene expression pattern was similar in mature hESC-RPE Regea08/017 cells compared to the human RPE cells (Figure S1). In addition, hESC-RPE cells were polarized as seen in the vertical confocal sections demonstrating protein expression of CRALBP (green, cellular retinaldehyde-binding protein) and bestrophin (red) (Figure 1b). In the brightfield micrograph (Figure 1c), it can be seen that the hESC-RPE cells had acquired cobblestone morphology and a high degree of pigmentation, which is characteristic for mature RPE cells. The polarization of hESC-RPE cells, localization of apical tight junctions and brush border, high order of organization of extracellular matrix and the localization of pigmented granules are visible in low resolution TEM (Figure 1d). The partial intermingling of CRALBP and bestrophin label indicates that hESC-RPE cells had not reached the status of full maturation.

### 2.2. Proteasomes and Autophagy Regulate Amount of Melanosomes

**Figure 2:**
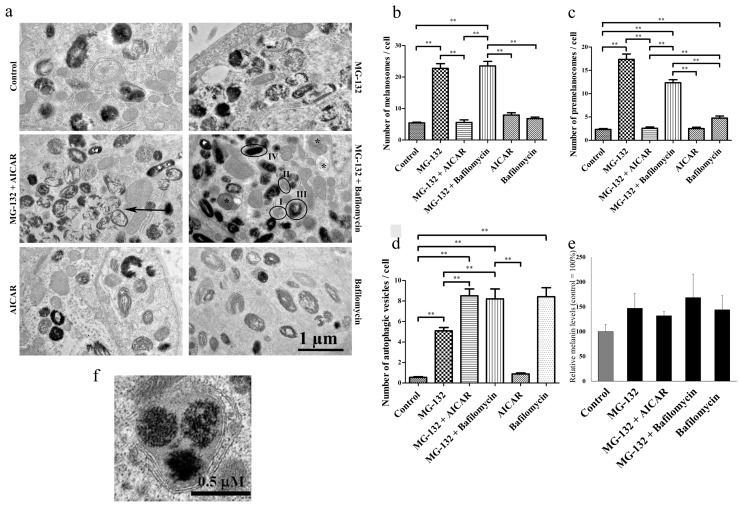
Representative transmission electron micrographs of hESC-RPE cells show that both autophagy and proteasomes regulate the amount of melanosomes after exposures to MG-132 (1 µM), AICAR (2 mM, 5-Aminoimidazole-4-carboxamide ribonucleotide) or/and bafilomycin A1 (50 nM) for 24 h. Control cells were exposed to culture medium (**a**). Quantification of melanosomes (**b**), premelanosomes (**c**) and autophagic vesicles (**d**) per 5-µm^2^ arbitrary selected areas per treatment. Experiments were repeated 3× independently. Proteasome inhibition by MG-132, as well as bafilomycin A1 exposure increase melanin levels in hESC-RPE cells. Simultaneous proteasome inhibition with AICAR decreases pigmentation. Melanin light absorbance at 690 nm was normalized with protein concentration of each individual sample. Results are means ± standard deviation (SD) relative to the control sample from two independent experiment. The melanin level set at 100% corresponds to the amount of 0.452 ± 0.07 µg melanin/µg protein in the sample (**e**). An example of the double membrane autophagosome around melanosomes in hESC- RPE cells treated with MG-132 and AICAR (**f**). ** *p* < 0.01, Mann–Whitney U. The arrow indicates a double membrane autophagosome, the asterisk a single membrane autophagolysosome and Roman numerals (I–IV) different stages of circuited melanosomes. Double membrane and/or degradative material inside of vesicles were criteria for phagosome calculation. Organelles were manually calculated from transmission electron microscopy (TEM) micrographs. Typical micrographs were selected for analysis. The TEM photographer knew the sample codes, but examiners were masked during organelle analysis.

The treatments and doses utilized in this work were chosen due to their known and validated actions on autophagic machinery. Proteasome inhibition, e.g., with MG-132, has been shown to activate autophagy [2,3,5,41]. AICAR (5-Aminoimidazole-4-carboxamide ribonucleotide), an analogue of adenosine monophosphate (AMP), can stimulate AMP-dependent protein kinase (AMPK) activity, inhibit mechanistic target of rapamycin (mTor) and then induce autophagy [3,42,43,44,45,46,47]. Bafilomycin A1 is an inhibitor of vacuolar type H^+^-ATPase, and while preventing the acidification of lysosomes, it also inhibits fusion of lysosomes with autophagosomes and formation of autolysosomes [42,43]. The effects of MG-132, AICAR, bafilomycin A1, MG-132 + AICAR or MG-132 + bafilomycin A1 for 24 h on the number and maturation of melanosomes and autophagosomes within hESC-RPE cells were visualized with TEM. Proteasome inhibition with MG-132 evoked accumulation of both premelanosomes and autophagosomes (*p* < 0.01; Figure 2). Autophagy inducer AICAR heavily increased the amount of autophagosomes, but decreased the number of premelanosomes during proteasome inhibition (MG-132 vs. AICAR + MG-132, *p* < 0.01). As expected, bafilomycin A1 treatment strongly increased the number of autophagosomes (Figure 2a,d,e). AICAR treatment did not show significant changes in the number of melanosomes, premelanosomes or autophagic vesicles. Thus, AICAR seems to accelerate autophagic process during proteasome inhibition. In addition, we observed that bafilomycin A1 rather than AICAR increased the number of melanosomes under proteasome inhibition (*p* < 0.05; Figure 2a,c). 

Melanin has a broad absorbance spectrum, which can be used for melanin quantitation [48]. In addition to microscopic evaluation of pigmentation in cells, melanin pigment levels were also quantitated from cell lysates by using absorbance spectroscopy at 690 nm. In accordance with the microscopy, the absorbance spectrum of MG-132-treated samples displayed increased melanin levels compared to control samples (Figure 2e), and it was even more pronounced together with bafilomycin A1. However, when the cells were exposed to MG-132 together with AICAR, a moderate change in melanin levels was observed. Note that the amount of melanin is in line with the number of melanosomes in different treatments, but statistical significance was not observed possibly due to the limited sample size (*n* = 2).

### 2.3. 5-Aminoimidazole-4-carboxamide Ribonucleotide Decreases Amount of Microtubule-Associated Protein 1A/1B-Light Chain 3 and Sequestosome-1 during Proteasome Inhibition

**Figure 3:**
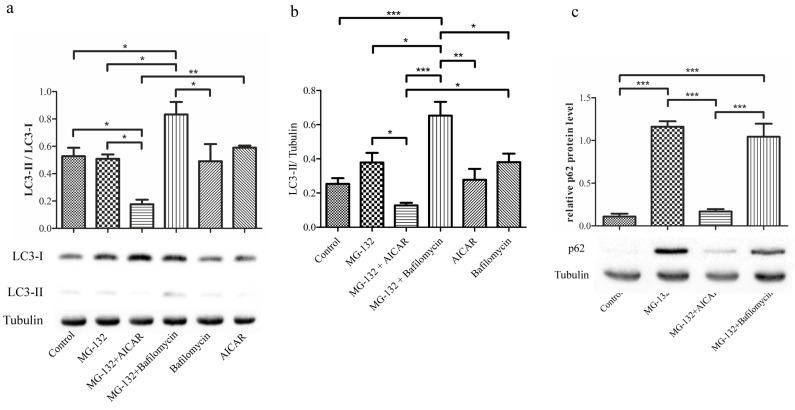

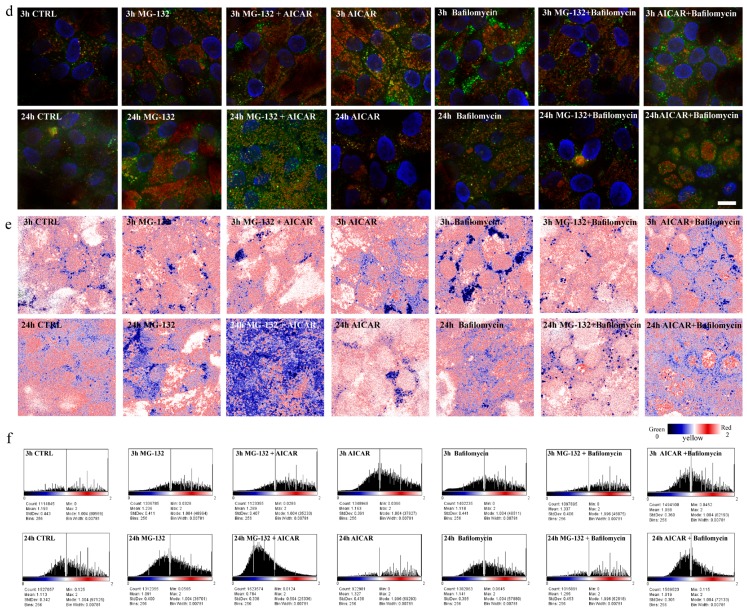
Representative Western blotting analysis and pH-sensitive Green Fluorescent Protein (GFP)-mCherry-LC3A vector shows that AICAR decreases protein levels of LC3–II/I (microtubule-associated protein 1A/1B-light chain 3) (**a**,**b**) and p62 (**c**) and induced autophagy flux (**d**–**f**) during proteasome inhibition. Proteins in the total homogenates of hESC-RPE cells after exposures to MG-132 (1 µM), AICAR (2 mM), or/and bafilomycin A1 (50 nM) for 24 h, or medium in control cells were analyzed using Western blot, with α-tubulin as a loading control. Statistical analysis was performed by ANOVA followed by Tukey’s test for multiple comparison, * *p* < 0.05, ** *p* < 0.01, *** *p* < 0.001. Representative confocal microscopy images (*n* = 3–4 biological replicates and in each 1–4 images/sample) of mature hESC-RPE cells 24 h after transfections with green fluorescent protein (GFP)-mCherry-LC3A, where AICAR treatment has induced autophagy flux with proteasome inhibition. (**d**) From left to right are: the control (CTRL), i.e., transfected sample without other treatments; transfected and treated with MG-132 (1 μM); transfected and treated with MG-132 and AICAR (2 mM); transfected and treated with AICAR (2 mM); transfected and treated with bafilomycin A1 (50 nM); transfected and treated with MG-132 (1 μM) and bafilomycin A1 (50 nM); transfected and treated with AICAR (2 mM) and bafilomycin A1 (50 nM) for 3 h or 24 h. Scale bar = 10 µm. (**e**) The area of green, yellow (autophagosomes) and red (autolysosomes) of the confocal images were analyzed with Image J by dividing pixels of red channel with the pixels of green channel; the output was re-scaled from 0–2 and presented with the UnionJack color scale (0 = black, white = 1 and red = 2). (**f**) In order to visualize the output better, the results are shown as histograms.

The functionality of the autophagic machinery was examined by assessing the amount of autophagy marker proteins p62, LC3-II and the ratio of LC3–II/I in Western blots of whole cell extracts. Conversion of the cytosolic form of LC3-I to the membrane-bound phosphatidylethanolamine (PE) lipidated LC3-II form indicates autophagic activity [49]. The p62 protein is usually found in protein aggregates with polyubiquitinated proteins, and when autophagosomal function is inhibited, the amount of p62 is usually increased [2,3,5]. The turnover, which is the degradation rate of LC3-II within autolysosomes, can be quantified when analyzing the amount of LC3-II after treatments [5]. The ratio of LC3-II/I was highest when cells were treated with a combination of proteasome inhibitor MG-132 and autophagy inhibitor bafilomycin A1 for 24 h (Figure 3a and Figure S6). MG-132 treatment slightly increased the level of LC3-II, but because the level of LC3-I was also increased by the treatment, the resulting LC3 ratio was similar to the control. AICAR treatment together with MG-132 decreased the level of LC3-II indicating activated autophagy (Figure 3b). Proteasome inhibition with MG-132 evoked an intensive accumulation of p62 (Figure 3c and Figures S2 and S7). In line with LC3 data, the combination treatment with MG-132 and AICAR abolished expression of p62 when compared to pure MG-132 treatment. Since p62 co-localizes with LC3 and AICAR enhances autophagy, it is reasonable to assume that increased autophagy has led to decreased levels of both p62 and LC3-II through increased degradation [5].

### 2.4. 5-Aminoimidazole-4-carboxamide Ribonucleotide Treatment Induces Autophagy Flux with Proteasome Inhibition

The fluorescently-tagged fusion proteins, green fluorescent protein (GFP)-mCherry-LC3A, pEGFP-LC3 and pDsRed2-hp62 plasmids (p), were analyzed with confocal microscopy after treatment with MG-132, AICAR, bafilomycin A1 alone or in combination. This pH-sensitive GFP-mCherry-LC3A vector emits green and red (yellow) fluorescence when at neutral pH (e.g., autophagosome), but emits only red fluorescence in acidic compartments (e.g., autolysosomes) because the fluorescence of GFP is quenched by the low pH. Cells transfected with GFP-mCherry-LC3A and treated with MG-132 showed induced formation of strongly red positive aggregates (Figure 3d), which is clearly visible also in the output image (Figure 3e) and histogram (Figure 3f). This is evidence that MG-132 induces the formation of acidic autolysosomes. The combination treatment with MG-132 and autophagy inducer AICAR evoked an intensive red color staining after a 3-h treatment (Figure 3e,f), which was obliterated after 24 h (Figure 3e,f), indicating effective autophagy flux. Green or yellow fluorescence was more prominent after treatment with AICAR, bafilomycin A1, AICAR + bafilomycin A1 or MG-132 + bafilomycin A1 and especially after MG-132 + AICAR, indicating the formation of autophagosomes, but not autolysosomes. GFP-mCherry-LC3A transfected cells exhibited noticeably less red color after AICAR + bafilomycin A1 treatment compared to MG-132 + AICAR treatment. Therefore, the combination treatment with AICAR + bafilomycin A1 was not considered to be an important control in further analyses used in this work.

The second transfections with both pEGFP-LC3 and pDsRed2-hp62 resulted in faint cytoplasmic fluorescence, observed using confocal microscopy, indicating that transfection of hESC-RPE cells was successful (Figure S3). The MG-132 treatment induced the formation of small pEGFP-LC3 and pDsRed2-hp62-positive aggregates. Meanwhile, formation of aggregates was not observed after the combination treatment with MG-132 and autophagy inducer AICAR. Incubation with bafilomycin A1 evoked increased double staining of the pEGFP-LC3 and pDsRed2-hp62 constructs (Figure S3).

### 2.5. Proteasome Inhibition and Bafilomycin A1 Induces Melanogenesis

The lightness of microscopic images has been previously shown to be an objective and quantitative method to evaluate the degree of RPE cell pigmentation [47]. Therefore, we acquired five phase contrast brightfield images with identical light exposure and illumination settings from randomly selected areas in all cell culture inserts and evaluated the darkness from each image. This pigmentation analysis indicates that exposure to MG-132 reduces the lightness of hESC-RPE cells (Figure S4). The changes were statistically significant compared to control after MG-132 treatment (*p* < 0.001) and similarly after MG-132 + bafilomycin A1 (*p* = 0.002) treatment. MG-132 significantly (*p* < 0.001) increased pigmentation also when comparing bafilomycin A1 and MG-132 + bafilomycin A1 treated samples. Finally, the decrease of pigmentation after AICAR treatment (MG-132 vs. MG-132 + AICAR) was highly significant (*p* < 0.001). 

**Figure 4:**
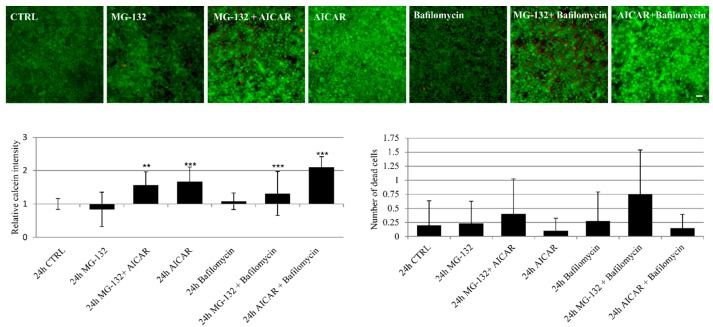
Representative micrograph images of the viability assay of mature hESC-RPE cells 24 h after exposures to MG-132 (1 µM), AICAR (2 mM) or/and bafilomycin A1 (50 nM) for 24 h compared to the control. Scale bar 20 µm. Underneath the micrographs are graphs where the intensity of calcein has been analyzed with Image J software and the number of dead cells (red nuclei), both from five fields of view. Statistical analysis was performed by two-tailed Mann–Whitney U against the control, ** *p* < 0.01, *** *p* < 0.001.

It is essential to verify that the used chemical treatments do not decrease cell viability or increase cell death. Here, it was assessed with a commercial viability assay. Microscopic observation revealed that the chemical treatments used in these experimental procedures altered the relative intensity of calcein-AM fluorescence compared to control: fluorescence was decreased in MG-132-treated samples, but increased in MG-132 + AICAR- and AICAR-treated samples (Figure 4). It should be noted that calcein fluorescence intensity is dependent both on cell viability and the amount of pigmentation. Higher fluorescence in MG-132 + AICAR and AICAR treatments reveals both increased autophagy and decreased pigmentation, while lower fluorescence in MG-132, MG-132 + bafilomycin A1 and bafilomycin A1 is associated with the melanosome number and melanogenesis (Figure 2a,c). There were no statistically-significant differences in the number of dead cells between treated samples and control (Figure 4).

## 3. Discussion

One of the most central characteristics of RPE cell phenotype is melanin pigmentation in lysosome-related organelles, called melanosomes [16]. Melanin eliminates ROS and acts as a chelator of metals, thus protecting young RPE cells [16,17,18]. However, cytoprotective effects are gradually lost during aging, leading to decreased antioxidant capacity and also vice versa to increased oxidative stress [19,20,21,22,23]. Melanosome differentiation and cellular pigmentation are controlled by proteins whose turnover is regulated by proteasomes [50,51,52,53]. Watabe et al. reported that tyrosinase activity and melanosome production are regulated by whether the protein is retained in the endoplasmic reticulum to be degraded by proteasomes or transported through Golgi and endosomes to premelanosomes [50]. The transport from endoplasmic reticulum is influenced by organelle pH. Proteasome inhibition has been shown to affect lysosomal degradation and endosomal sorting of membrane proteins to lysosomes [54,55]. Thus, exposing cells to MG-132 and bafilomycin A1 can be anticipated to regulate tyrosinase processing, trafficking and melanogenesis [49]. Inhibition of proteasomes may result in reduced proteasomal degradation of tyrosinase and therefore increased levels of tyrosinase and premelanosomes as observed in the present study. Previous findings indicate that also autophagy takes part in the regulation of cellular pigmentation [28,29,30,31].

Autophagic processes have been assessed previously using the immortalized ARPE-19 RPE cell line [3,5,49]. Nonetheless, hESC-RPE cells differ from ARPE-19 cells in their gene expression [35] and pigmentation [56], as well as aggregation levels under proteasome inhibition [2,3,5]. In this study, we show that proteasome inhibition leads to high accumulation of melanosomes in hESC-RPE cells, a finding in line with previous documentations [28,52,53,57]. The lower calcein fluorescence intensity detected in live/dead viability assay is in line with the accumulation of melanosomes in MG-132- and MG-132 + bafilomycin A1-treated samples. This may be cellular compensatory mechanism to provide cytoprotection via melanin [18,28,52,53]. Functional autophagy can be interpreted as a beneficial effect for preventing RPE degeneration [49,58,59]. Cleansing of lipofuscin loaded melanosomes in autophagy may be anticipated to decrease oxidative stress in aged RPE cells [22,23,26]. In young RPE cells, many other cytoprotective systems, such as antioxidant production, melanogenesis, molecular chaperone response, balanced proteolysis and DNA damage response may compensate autophagy [18,25,48,60,61,62,63]. Autophagy regulating proteins have also been shown to accelerate melanogenesis that support beneficial dual role of autophagy in the regulation of melanosome turnover [31]. Autophagy provides selective clearance and participation in exocytosis [64], but its role in clearance of lipofuscin loaded melanosomes is not known. 

In this study, our observations revealed that autophagy cleanses melanosomes under proteasome inhibition. This was also seen here as increased calcein fluorescence in the live/dead assay. Moreover, inhibition of the basal autophagosome flux level by bafilomycin A1 induced melanogenesis in hESC-RPE cells, suggesting crosstalk of proteasomes and autophagosomes in the regulation of pigmentation in this cell type. When the autophagic process is impaired in ARPE-19 cells, they develop strong perinuclear aggregation, but weak pigmentation [3,5]. In contrast, hESC-RPE cells indicated high pigmentation levels, but a relatively weak protein aggregation. In line with our recent publications [3,5], the autophagy receptor p62 also accumulated in response to proteasome inhibition in hESC-RPE cells. However, inhibition of the autophagosome-lysosome fusion with bafilomycin A1 did not increase the level of p62 in mature hESC-RPE cells, as has been previously shown in ARPE-19 cells [5]. Formerly, autophagy clearance was revealed by using ARPE-19 cells in which the amount of perinuclear protein aggregates of p62 and LC3-II was downregulated during proteasome inhibition and AICAR exposure [5]. In this study, Western blot analysis exhibited an upregulation of p62 during proteasome inhibition that was alleviated when combined with AICAR treatment. Moreover, autophagy flux was confirmed with confocal microscopy by using a GFP-mCherry-LC3A vector.

## 4. Materials and Methods

### 4.1. Cell Culture and Treatments

The pluripotent hESC line Regea08/017 was derived and characterized in our laboratory at the University of Tampere, Finland, as described previously [39]. The hESC line was derived and cultured on top of mitotically inactivated (either γ-irradiated, 40 Gy or mitomycin C (10 μg/ mL, Sigma-Aldrich, St. Louis, MO, USA) treated) human foreskin fibroblast feeder cells (CRL-2429TM, ATCC, Manassas, VA, USA). The hESCs were cultured at 37 °C and 5% CO_2_ with hESC culture medium consisting of Knock-out Dulbecco’s Modified Eagle Medium (KO-DMEM) supplemented with 20% Knock-Out Serum Replacement (KO-SR), 2 mM Glutamax-I, 0.1 mM 2-mercaptoethanol (all from Life Technologies, Carlsbad, CA, USA), 1% non-essential amino acids (NEAA), 50 U/mL penicillin/streptomycin (both from Lonza Group Ltd., Basel, Switzerland) and 8 ng/mL human basic fibroblast growth factor (bFGF) (Peprotech, London, UK). The culture medium was replenished 5× a week, and undifferentiated colonies were manually passaged onto new feeder cells once a week. 

Undifferentiated hESCs were induced to differentiate into RPE cells in floating cell aggregates by lowering the KO-SR concentration to 15% and removing bFGF, as previously described [34]. Floating aggregates were cultured for 76–140 days. The culture medium was replenished 3× a week. The pigmented areas of floating aggregates were manually dissected, dissociated with 1× trypsin-EDTA (ethylenediaminetetraacetic acid) and replated on collagen IV from human placenta (5 μg/cm^2^; Sigma-Aldrich) for enrichment of cell number. 

After 108–322 days of culture, the pigmented areas were enzymatically dissociated with 1× trypsin-EDTA and seeded at a density of 100,000 cells/cm^2^ either on 24-well plates (BD Biosciences, San Jose, CA, USA) or PET cell culture inserts (Millipore Corporate, Billerica, MA, USA) coated with collagen IV (Sigma-Aldrich, St. Louis, MO, USA). The cells were further cultured for 80–194 days (a total of 158–356 days of culture). According to our standard procedure, the cultures were used for experiments when they have reached transepithelial electrical resistance (TEER) above 100 Ω·cm^2^, but preferentially over 200 Ω·cm^2^. The cell culture periods and number of replicates are presented in Table 1 and passage numbers and culture periods of independent experiments in Table S1. 

Mature hESC-RPE cells were treated with 1 μM MG-132 (Calbiochem, San Diego, CA, USA), 2 mM AICAR (AICA ribonucleotide, 5-aminoimidazole-4-carboxamide-1-β-d-ribofuranoside, Toronto Research Chemical, North York, ON, Canada, A611700), 50 nM bafilomycin A1 (Sigma-Aldrich) alone or in combination: 1 μM MG-132 + 50 nM bafilomycin A1 and 1 µM MG-132 + 2 mM AICAR (Figure S8). The control cells were treated with cell culture medium with the KO-SR of 15%, as described earlier. All treatments were carried out for 3 h and/or 24 h at 37 °C and 5% CO_2_.

### 4.2. Immunofluorescence Labelling

Adherent cells were subjected to immunofluorescence (IF) staining to detect the localization of RPE cell specific proteins as previously described [26]. Briefly, the cells were washed 3 × 5 min with PBS, fixed 10 min with 4% paraformaldehyde (pH 7.4; Sigma-Aldrich), washed with PBS, permeabilized in 0.1% Triton X-100/PBS (Sigma-Aldrich) for 10 min and washed 3× with PBS. Non-specific binding sites were blocked with 3% BSA (Sigma-Aldrich) in PBS for 1 h. Primary antibody incubations (Table 2) were carried out in 0.5% bovine serum albumin (BSA)-PBS for 1 h. Thereafter, cells were washed 3× with PBS. Secondary antibody incubations (Table 2) were carried out in 0.5% BSA-PBS for 1 h. Cells were washed 3× with PBS. Nuclei were counterstained with DAPI in VectaShield mounting medium (Vector Laboratories Inc., Burlingame, CA, USA). The entire labelling procedure was performed at room temperature (RT). Brightfield images were obtained with Zeiss AxioScope A1 phase contrast microscope with 63× magnification (Carl Zeiss, Jena, Germany). Confocal microscopy images were obtained with an LSM 700 confocal microscope (Carl Zeiss, Jena, Germany) using a 63× oil immersion objective. Overlays and image processing of confocal images were done in ZEN-software (Carl Zeiss). The negative control cells were cultured, stained and visualized similarly as actual immunofluorescence samples, except that the primary antibodies were omitted. Used antibodies are presented in Table 2.

### 4.3. RNA Extraction, Complementary DNA Synthesis and Reverse Transcription-Polymerase Chain Reaction

In order to analyze the gene expression of mature hESC-RPE cells, total RNA was extracted from hESC-RPE cells with a NucleoSpin XS-kit (Macherey-Nagel, GmbH & Co., Düren, Germany) according to the manufacturer’s instructions. The RNA concentration and its quality were assessed using NanoDrop 1000 spectrophotometer (NanoDrop Technologies, Wilmington, DE, USA). RNA (40 ng) was reverse-transcribed to complementary DNA using MultiScribe Reverse Transcriptase (Applied Biosystems, Foster City, CA, USA) according to the manufacturer’s instructions in the presence of an RNase inhibitor. Complementary DNA (cDNA) was used as a template in a PCR reaction, which was carried out using 5 U/μL Taq DNA Polymerase (Fermentas, Thermo Fisher Scientific Inc., Leicester, UK) with 5 μM specific primers (Biomers.net GmbH, Söflinger, Ulm, Germany, Table 3). The PCR reactions were carried out in PCR MasterCycler ep gradient (Eppendorf AG, Hamburg, Germany) as follows: 95 °C 3 min, 95 °C 30 s, annealing 30 s, 72 °C 1 min, 72 °C 5 min, for 38 cycles. Annealing temperatures and primer sequences are presented in Table 3 and Figure S1. PCR products were resolved in 2% agarose gels with a 50-base pair DNA ladder (MassRulerTM DNA Ladder Mix, Fermentas). The bands were visualized with the Quantity one 4.5.2. Basic program (Bio-Rad Laboratories, Inc., Hercules, CA, USA).

### 4.4. Transmission Electron Microscopy

For transmission electron microscopic (TEM) examination, hESC-RPE cell samples were fixed with 2% glutaraldehyde (Electron Microscopy Sciences, Hatfield, PA, USA) prepared in 0.1 M phosphate buffer for 2 h at RT followed by incubation in 0.1 M phosphate buffer overnight at room temperature. Thereafter, the samples were post-fixed with 1% osmium tetroxide (Ladd Research, Williston, VT, USA) for 1 h at RT and dehydrated through a graded series of acetone (J. T. Baker, Avantor Performance Materials B.V., Deventer, The Netherlands): 70% acetone, 94% acetone and absolute acetone. The samples were then infiltrated in a 1:1 mixture of absolute acetone and epoxy resin (Ladd Research) for 1.5 h at RT, embedded in pure epoxy resin overnight at RT and polymerized for 48 h at 60 °C. Thin sections were stained with 1% uranyl acetate for 30 min and with 0.4% lead citrate (Fluka, Steinheim, Germany) for 5 min. Samples were examined and imaged with the JEM-2100F transmission electron microscope (TEM, JEOL Ltd., Tokyo, Japan). Identification of premelanosomes and melanosomes were done as shown previously [65].

### 4.5. Melanin Quantitation by Absorption Spectroscopy

Melanin pigment in the cell samples lysed in M-PER (Mammalian Protein Extraction Reagent) lysis buffer (see Section 4.6) was pelleted by centrifugation at 16,000× *g* for 17 min. After removal of supernatant, pigmented pellets were re-suspended in 100 µL of fresh M-PER buffer and absorbance was measured at 690 nm in clear 96-well plates by using EnVision multi-label reader (Perkin Elmer, Waltham, MA, USA). The RPE-choroid melanin isolated from porcine eyes was used as a standard [40].

### 4.6. Western Blot

The cell samples were washed with PBS (Lonza Group Ltd.), and cells were lysed in M-PER lysis buffer (Thermo Scientific, Waltham, MA, USA) according to the manufacturer’s instructions 24 h after adding the chemical treatments. Whole cell extracts (25–30 μg of protein) were run in 15% sodium dodecyl sulfate polyacrylamide gel electrophoresis (SDS–PAGE) gels and thereafter wet-blotted to nitrocellulose membranes (Amersham, Pittsburgh, PA, USA). The blocking of membranes was done in 3% skimmed milk powder in 0.3% Tween-20/PBS at room temperature for 1.5 h. Thereafter, the membranes were incubated with mouse monoclonal p62 antibody (Cat. No. sc-28359; Santa Cruz Biotechnology Inc., CA, USA) diluted 1:1000 in 0.5% BSA in 0.3% Tween-20/PBS, rabbit polyclonal LC3 antibody (Cat. No. 3868; Cell Signaling, Danvers, MA, USA) diluted 1:1000 in 5% BSA in 0.1% Tween-20/TBS (Tris-buffered saline) overnight at 4 °C, or mouse monoclonal α-tubulin antibody (Cat. No. T5168, Sigma-Aldrich) diluted 1:8000 in 1% milk powder in 0.05% Tween-20/PBS for 1 h at RT. After washing 3× for 5 min with 0.3% Tween-20/PBS (p62 antibody), 0.1% Tween-20/TBS (LC3 antibody) or 0.05% Tween-20/PBS (alpha-Tubulin), the membranes were incubated for 2 h at RT (except for alpha-Tubulin, which was incubated for 1 h) with horseradish peroxidase-conjugated anti-rabbit IgG (Novex™, #A16014, Thermo Fisher Scientific, Rockford, IL, USA) or anti-mouse Imunoglobulin G (IgG) antibodies (GE Healthcare, Little Chalfont, UK). The secondary antibodies were diluted 1:10,000 in 3% skimmed milk powder in 0.3% Tween-20/PBS for p62, 3% skimmed milk powder in 0.1% Tween-20/TBS for LC3 and 1% skimmed milk in 0.05% Tween-20/PBS for α-tubulin. Before detection, all membranes were washed as described earlier. Protein–antibody complexes were detected with an enhanced chemiluminescent assay for horseradish peroxidase (Millipore, Billerica, MA, USA).

### 4.7. Confocal Microscopy Analysis

Mature hESC-RPE cells with a cobblestone morphology were transfected with a tandem fluorescently tagged LC3 (GFP-mCherry-LC3A), which at neutral pH fluoresces yellow in autophagosomes. Acidification quenches the GFP signal, thus only the red fluorescence of mCherry is detected in autolysosomes [8]. The transfection protocol is described previously [66]. Briefly, 250 μg of GFP-mCherry-LC3A were added to ExGen500 transfection reagent (Fermentas, Thermo Fisher Scientific Inc., Leicester, UK) following the manufacturer’s instructions. Transfection was continued for 24 h at 37 °C and 5% CO_2_. Thereafter, cells were exposed to MG-132, AICAR and/or bafilomycin A1 for 3 h or 24 h, then mounted in Vectashield with DAPI (H-1200) and placed on ice. Samples were examined immediately with LSM 700 confocal microscope 63× oil immersion similarly as described for immunofluorescence samples in Section 2.2. The amount of red, green and yellow of the confocal images was analyzed with Image J. The pixels of red channel were divided with the pixels of green channel, re-scaled from 0–2 (green equalized 0; yellow = 1 and red 2. The output was presented with UnionJack color scale (0 = black, white = 1 and red = 2) to enhance the visibility. In addition, the separation is shown in histograms. Additional transfections were performed with plasmids pEGFP-hLC3Am and pDsRed2-hp62 (Figure S3).

### 4.8. Cell Viability

The effects of 1 μM MG-132, 50 nM bafilomycin A1, 2 mM AICAR or their combination treatments on cell viability were assessed using Live/Dead Viability/Cytotoxity kit for mammalian cells (Invitrogen, Carlsbad, CA, USA). Briefly, 23.5 h after commencing the chemical treatments, the cells were rinsed with HEPES-buffered salt solution (HBSS, Lonza Group Ltd., Basel, Switzerland). Thereafter, cells were incubated for 30 min at 37 °C and 5% CO_2_ with a mixture of 0.25 µM calcein-AM (green fluorescence) and 0.5 µM Ethidium homodimer-1 (EthD-1, red fluorescence) diluted in HBSS, either in the presence or absence of chemicals. Cells were imaged under a fluorescence microscope (Olympus IX) to detect the viable (green fluorescence) and dead cells (red fluorescence) with a 20× long working distance objective. The same imaging acquisition settings were applied for all samples. The calcein-AM fluorescence intensity was quantified with Image J software from 5 separate 288 × 288 dpi areas (which contained 62–88 cells) from images of two separate experiments. The intensities were correlated to the untreated control. The number of dead cells (red fluorescence) was also calculated from 5 separate 288 × 288 dpi areas from images of two separate experiments.

### 4.9. Ethical Issues

BioMediTech at University of Tampere has the approval of the National Authority for Medicolegal Affairs Finland to study human embryos (Diary number1426/32/300/05), and a supportive statement of the Ethical Committee of the Pirkanmaa Hospital District to derive, culture and differentiate hESC lines from surplus human embryos (R05116). No new lines were derived for this study. All methodological details were carried out in accordance with the approved guidelines.

### 4.10. Statistical Analyses

The statistical significance of quantification of melanosomes, premelanosomes and autophagic vesicles, relative calcein-AM intensity and number of dead cells in the field of view and the degree of pigmentation analyzed from brightfield images were analyzed with PASW Statistics (Version 18, Quarry Bay, Hong Kong, China), using the two-tailed Mann–Whitney U test. The data from Western blot experiments were subjected to one-way analysis of variance (ANOVA), followed by Tukey’s test for multiple comparison (*p*-values < 0.05 were considered significant). The number of replicates in each experiment is indicated in the figure legends.

## 5. Conclusions 

In this work, we showed how proteasomal inhibition increased the amount of highly pigmented melanosomal granules and the accumulation of p62 in hESC-RPE cells. Furthermore, the AMPK activator AICAR promoted cleansing of melanin granules, p62 and LC3-II during proteasome inhibition. These data indicate that autophagy machinery is functional in hESC-RPE cells and may have a role in the regulation of cellular pigmentation. 

## Figures and Tables

**Table 1:** Culture periods of cell samples.

Analysis	Adherent Culture Period (SD)	Entire Culture Period (SD)
RT-PCR (*n* = 4)	d104 (83–112)	d230 (235–244)
TEM (*n* = 4)	d127 (36–194)	d279 (158–404)
WB (*n* = 8)	d100 (51–194)	d299 (244–383)
IF (*n* = 79)	d83 (36–112)	d277 (197–356)
Live-dead (*n* = 2)	d83 (36–112)	d277 (197–356)

TEM: Transmission Electron Microscopy; WB: Western blot; IF: immunofluorescence.

**Table 2:** Antibodies used in immunofluorescence labelling.

Antibody Name	Abbreviation	Primary/Secondary	Host	Dilution	Cat	Manufacturer
Anti-cellular retinaldehyde-binding protein	CRALBP	primary	mouse monoclonal	1:1000	ab15051	Abcam, Cambridge, UK
Rabbit anti-bestrophin	Bestrophin	primary	rabbit monoclonal	1:500	ab14928	Abcam
Anti-mouse IgG Alexa Fluor 488	-	secondary polyclonal	donkey	1:800	A21202	Molecular Probes, Life Technologies, Paisley, UK
Anti-rabbit IgG Alexa Fluor 568	-	secondary polyclonal	goat	1:800	A11011	Molecular Probes, Life Technologies

**Table 3:** Reverse-transcriptase-PCR primer sequences and used annealing temperatures.

Gene	Primer Sequences (5′ > 3′)		Tm
	Forward	Reverse	
*GAPDH*	GTT CGA CAG TCA GCC GCA TC	GGA ATT TGC CAT GGG TGG A	55
*OCT 3/4*	CGTGAAGCTGGAGAAGGAGAAGCTG	AAGGGCCGCAGCTTACACATGTTC	55
*PAX6*	AAC AGA CAC AGC CCT CAC AAA CA	CGG GAA CTT GAA CTG GAA CTG AC	60
*MITF*	AAG TCC TGA GCT TGC CAT GT	GGC AGA CCT TGG TTT CCA TA	52
*Bestrophin*	GAATTTGCAGGTGTCCCTGT	ATCAGGAGGACGAGGAGGAT	60
*RPE65*	TCC CCA ATA CAA CTG CCA CT	CAC CACC ACA CTC AGA ACT A	52

Tm: Annealing temperature.

## Supplementary Materials

Supplementary materials can be found at www.mdpi.com/1422-0067/18/5/1089/s1.
