# Supplementary material for: Autophagy Regulates Proteasome Inhibitor-Induced Pigmentation in Human Embryonic Stem Cell-Derived Retinal Pigment Epithelial Cells
Source: Int J Mol Sci. 2017 May 19;18(5):1089. doi: 10.3390/ijms18051089 (PMC5454998; doi:10.3390/ijms18051089)
Supplement: Supplementary file 1 [file ijms-18-01089-s001.pdf]

Article

Autophagy regulates proteasome inhibitor-induced pigmentation in human embryonic stem cell-derived retinal pigment epithelial cells

Kati Juuti-Uusitalo [1], Ali Koskela [2], Niko Kivinen [2], Johanna Viiri [2], Juha M.T. Hyttinen [2], Mika Reinisalo [3], Arto Koistinen [4], Hannu Uusitalo [5], Debasish Sinha [6], Heli Skottman [1], Kai Kaarniranta [2,7,*]

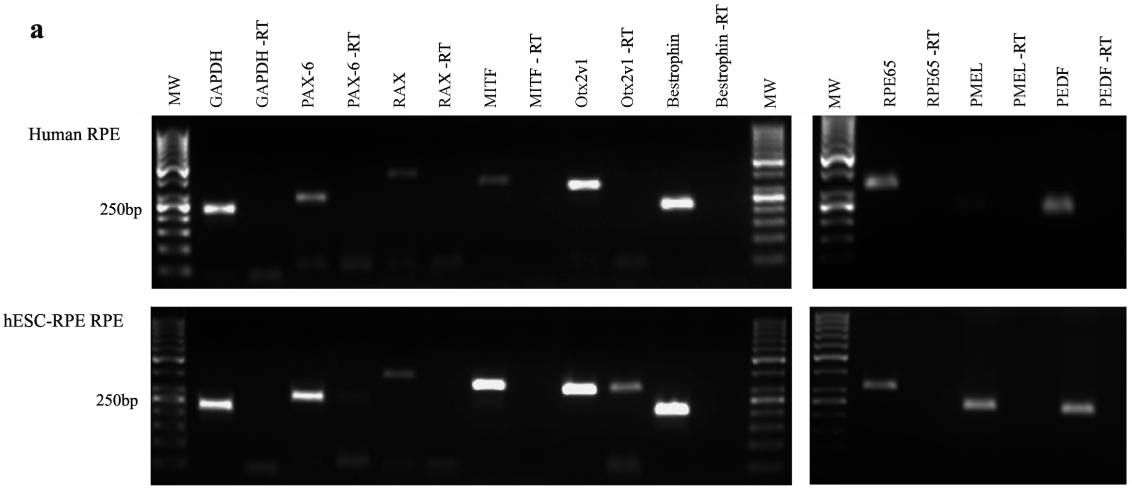

b

Gene	Forward	Reverse	Tann
	Primer sequences (5' > 3')		
GAPDH	GTT CGA CAG TCA GCC GCA TC	GGA ATT TGC CAT GGG TGG A	55
PAX6	AAC AGA CAC AGC CCT CAC AAA CA	CGG GAA CTT GAA CTG GAA CTG AC	60
RAX	CTG AAA GCC AAG GAG CAC ATC	CTC CTG GGA ATG GCC AAG TTT	55
MITF	AAG TCC TGA GCT TGC CAT GT	GGC AGA CCT TGG TTT CCA TA	52
OTX2v1	GGG CCC TGG GCT TCT TGT CC	ATT GGC CAC TTG TTC CAC TC	52
bestrophin	GAA TTT GCA GGT GTC CCT GT	ATC CTC CTC GTC CTC CTG AT	55
RPE65	TCC CCA ATA CAA CTG CCA CT	CAC CACC ACA CTC AGA ACT A	52
PMEL	GTG GTC AGC ACC CAG CTT AT	GAG GAG GGG GCT ATT CTC AC	52
PEDF	AGC TCG CCA GGT CCA CAA AG	TGG GCA ATC TTG CAG CTG AG	60

RT-PCR Primer sequences

Figure S1: The comparison of gene expression profiles between the fetal human RPE and the Regea08/017 hESC-RPE cell line. The comparison of gene expression profiles between the fetal human RPE and Regea08/017 hESC-RPE cell line. The human fetal RPE is a kind gift from School of Pharmacy (University of Eastern Finland). The cells and mRNA was extracted as described in Reinisalo M. et al., Mol. Vis. 2012. The hESC-RPE differentiated, mRNA extracted as described in the materials and methods. The rt-PCR, agarose gel and analysis was done similarly as described in the materials and methods. The PCR products visualized from agarose gels (a). The PCR product from the specific primer sequence is placed adjacent to –rt –negative control (i.e. sample which has not been treated with reverse transcriptase). The primer sequences and annealing temperatures are in the table (b).

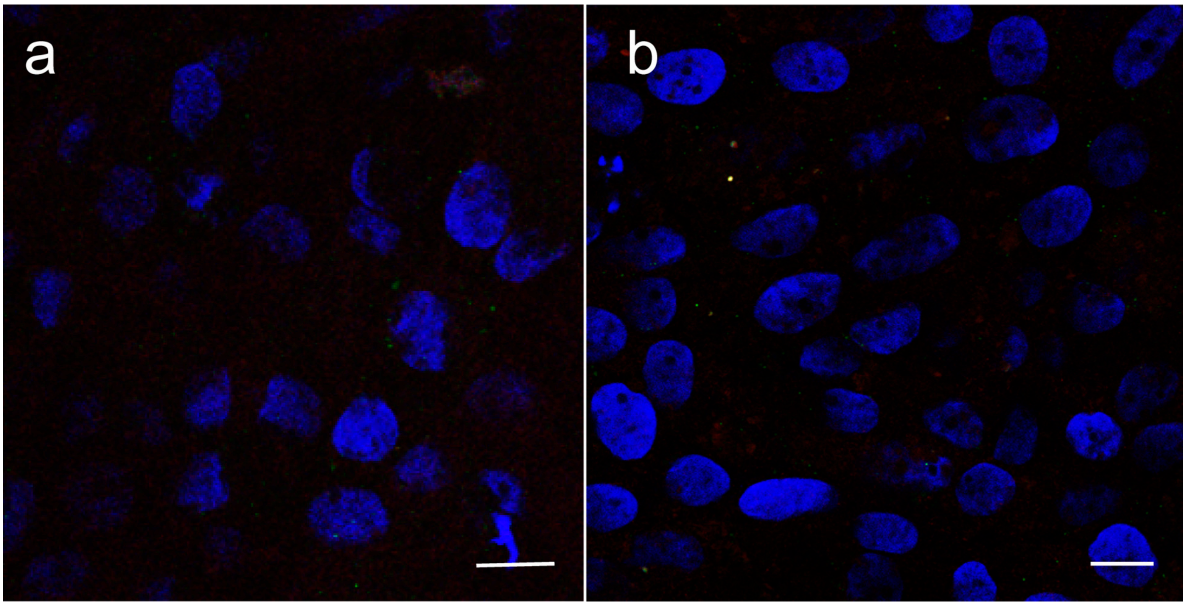

Figure S2: The negative immunofluorescence staining controls. In (a) the anti-mouse IgG Alexa Fluor 488 secondary antibody staining, and in (b) anti-rabbit IgG Alexa Fluor 568 staining. Scale bar 10 µm. Samples were treated and microscoped exactly as the actual immunofluorescenently labelled samples except that no primary antibodies were used, and the secondary antibodies were used individually. For the labelling, the cells were washed 3x5 min with 1x PBS, fixed 10 min with 4% paraformaldehyde (pH 7.4; Sigma-Aldrich, St. Louis, MO, USA), washed with PBS, permeabilized in 0.1% Triton X-100/PBS (Sigma-Aldrich) for 10 min, and washed 3x with PBS. Non-specific binding sites were blocked with 3% BSA (Sigma-Aldrich) in PBS for 1h. The primary were substituted with 0.5% BSA-PBS for 1h. Thereafter, cells were washed 3x with PBS. The secondary antibody staining, anti-mouse IgG Alexa Fluor 488 or anti-rabbit IgG Alexa Fluor 568, were carried out in 0.5% BSA-PBS for 1h. Cells were washed 3x with PBS. Nuclei were counterstained with DAPI (Vector Laboratories Inc., Burlingame, CA). The entire labelling procedure was performed at room temperature. Confocal microscopy images were obtained with an LSM 700 confocal microscope (Carl Zeiss, Jena, Germany) using 63x immersion objective. Overlays and image processing of confocal images were done in ZEN-software (Carl Zeiss).

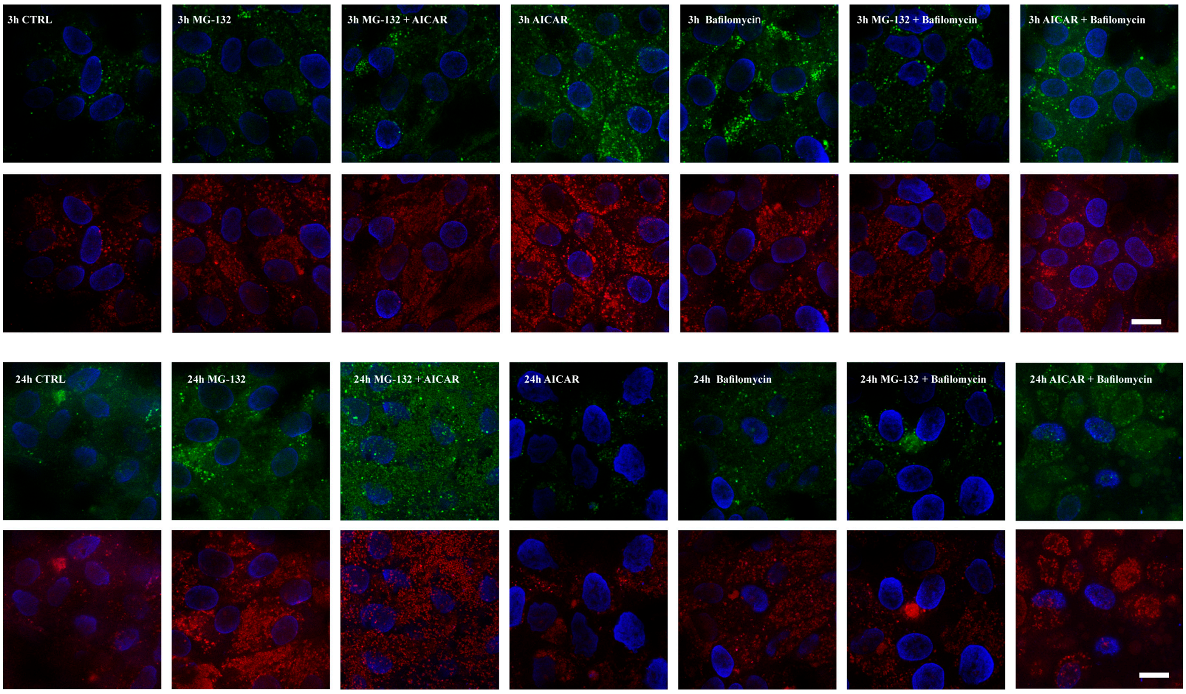

Figure S3: Confocal micrographs of mature hESC-RPE cells 3 and 24 h after transfections with GFP-mCherry-LC3A; there, the red and green channels are presented separately. Representative confocal microscopy images of mature hESC-RPE cells after 24h after transfections with GFP-mCherry-LC3A. The red and green channels are presented in separate images with the nuclear label DAPI (blue). From left to right are: Control (CTRL) i.e. transfected sample without other treatments; transfected and treated with MG-132 (1 µM); transfected and treated with MG-132 and AICAR (2 mM); treated and transfected with AICAR (2 mM); transfected and treated with bafilomycin A1 (50 nM); transfected and treated with MG-132 (1 µM) and bafilomycin A1 (50 nM); transfected and treated with AICAR (2 mM) and bafilomycin A1 for 3 and 24h. Samples were visualized without fixation immediately after finishing the experiment with an LSM 700 confocal microscope (Carl Zeiss, Jena, Germany) using a 63x oil immersion objective. Scale bar = 10 µm.

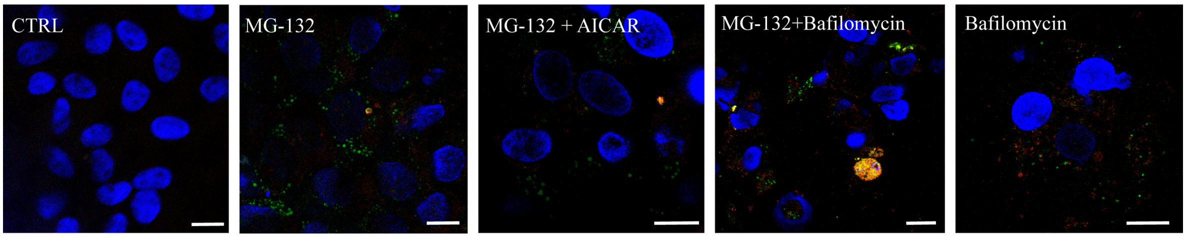

Figure S4: Confocal micrographs of mature hESC-RPE cells 48h after transfections with pEGFP-LC3 (green) and pDsRed2-hp62 (red). The preparation of the plasmid pDsRed2-hp62, producing the fusion protein of DsDeds red fluorescent protein and human SQSTM1/p62, has been previously described in Viiri J et al Mol Vis. 2010;16:1399-414. The plasmid pEGFP-hLC3A, producing the fusion protein of fluorescent green EGFP and human LC3A was constructed as follows. Human MAP1LC3A/LC3 (human microtubule-associated light chain 3A; NCBI gene bank no. AF303888) was amplified from DNase-treated (DNase I, Roche) total RNAextracted (Eurozol reagent, Euroclone) from human ARPE-19 retinal cells. Initially, mRNA was reverse-transcribed (MultiScribe reverse transcriptase, Applied Biosystems), and the LC3A open reading frame (ORF) was amplified with a high-fidelity DNA polymerase (Phusion Hot start DNA polymerase, Finnzymes). The following

primers were used: sense 5'-ATA CTCGAG at ATG CCG TCG GAG AAG A and reverse 5'-TGT AAG CTT g TTA CAC TGA CAA TTT CAT CCC. The restriction sites for XhoI and HindIII are in italics. The translation initiation and termination sites are in boldface. The additional bases enabling in-frame cloning are in minuscules. The sticky ends for the amplified LC3A ORFs as well as for the multiple cloning site of the vector pEGFP (Clontech) were produced with restriction endonucleases XhoI and Hind III (MBI Fermentas). Ligated (T4 DNA Ligase, Roche) DNA was transfected into competent DH5α E. coli cells. The integrity of the construct, named hereafter as pEGFP-hLC3A), was determined initially by restriction endonuclease digestion analysis and finally by sequencing of both the junction sites and the entire inserted MAP1LC3A/LC3 ORF. From left to right are: the control i.e. transfected sample without other treatments; transfected and treated with MG-132 (1µM); transfected and treated both with MG-132 and AICAR (2mM), transfected and treated with MG-132 and bafilomycin A1 (50 nM), transfected and treated with AICAR and transfected and treated with bafilomycin A1. Scale bar 10 µm.Confocal microscopy after the transfection with pEGFP-LC3 and pDsRed2-hp62 exhibited faint cytoplasmic fluorescence indicating that the transfection of hESC-RPE cells was successful. The MG-132 treatment induced formation of small pEGFP-LC3 and pDsRed2-hp62 positive aggregates but the combination treatment both with MG-132 and autophagy inducer AICAR obliterated the formation of aggregates. The incubation with bafilomycin A evoked increased merge staining of the pEGFP-LC3 and pDsRed2-hp62 constructs.

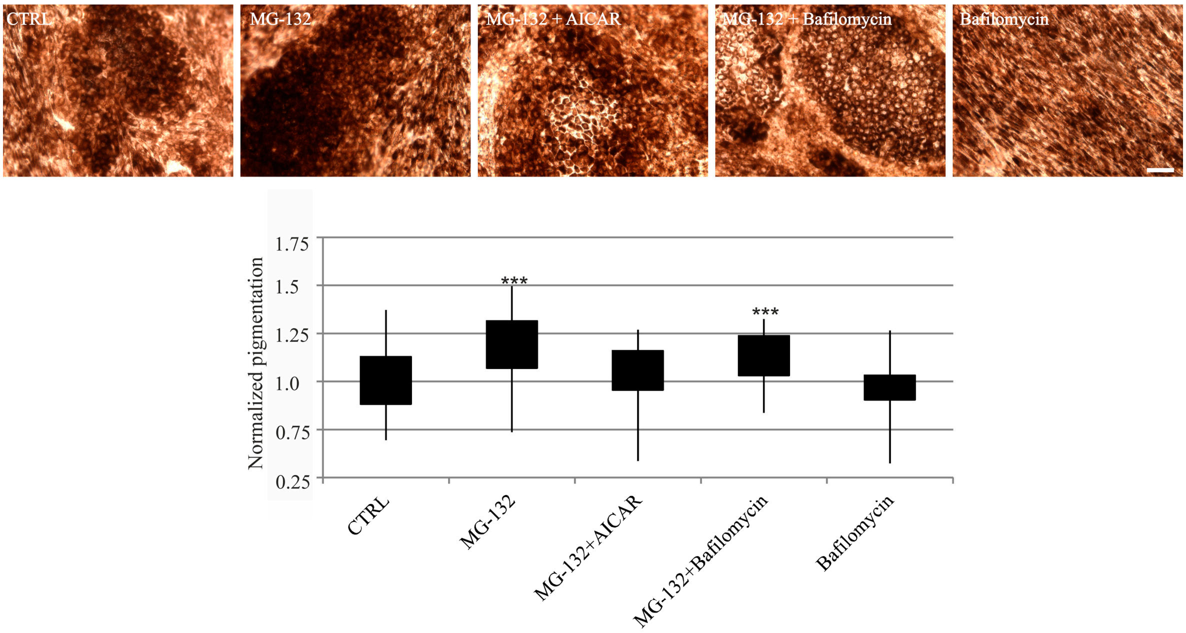

Figure S5: The degree of pigmentation of hESC-RPE cells was analyzed after exposures to MG-132 (1 µM), AICAR (2 mM) or/and bafilomycin A1 (50 nM) for 24 h, or medium in control cells by capturing 5 phase contrast bright field images from randomly selected locations on cell culture inserts with Zeiss Axioscope A1 with 20x objective. The light exposure settings and illumination were maintained constant between the samples. The degree of pigmentation was quantitated with Image J Image Processing and Analysis Software (http:// imagej.nih.gov/ij/index.html) through pixel intensity normalization. Inverse of pixel intensities was calculated in order to illustrate the density of pigmentation by subtracting the normalized pixel intensity from the maximum pixel intensity value for 8-bit grayscale images (similarly as in Sorkio et al. 2014). The inverse pixel intensities in each experiment were normalized against the control to equalize the variation between the experiments. Above are the representative phase contrast bright field images and below are analysed the box plot data showing the 1st and 3rd quartiles, and the range of the data.

Data from two biological and from those five technical replicates. The statistical analysis was performed by 2-tailed Mann-Whitney U against the control, ***$p<0.001$. Scale bar 50mm.

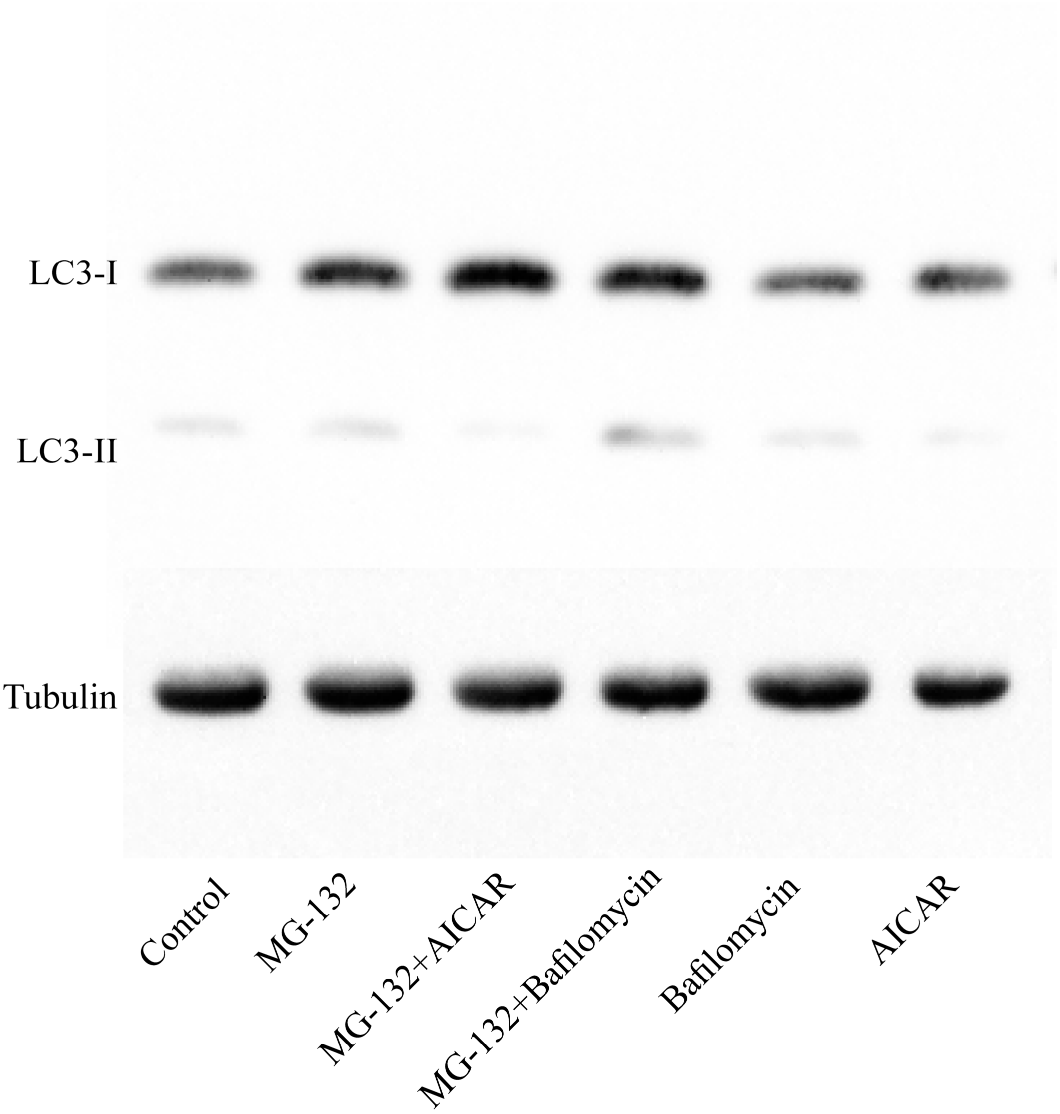

Figure S6: Original Western blot of LC3 from the total homogenates of hESC-RPE cells after exposures to MG-132 (1 µM), AICAR (2 mM) or/and bafilomycin A1 (50 nM) for 24h, or medium in control cells.

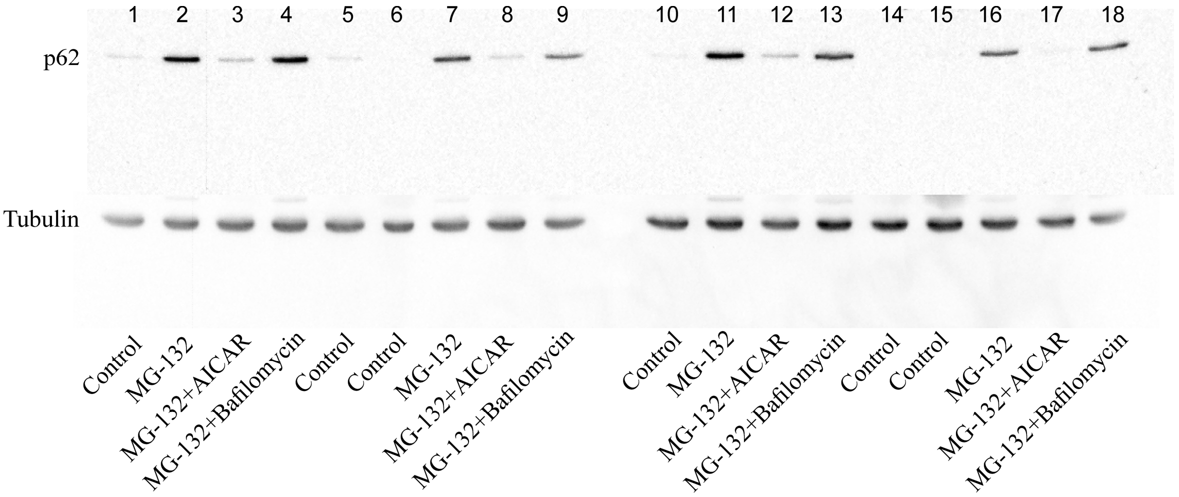

Figure S7: Original Western blot of p62 from the total homogenates of hESC-RPE cells after exposures to MG-132 (1 µM), AICAR (2 mM) or/and bafilomycin A1 (50 nM) for 24h, or medium in control cells.

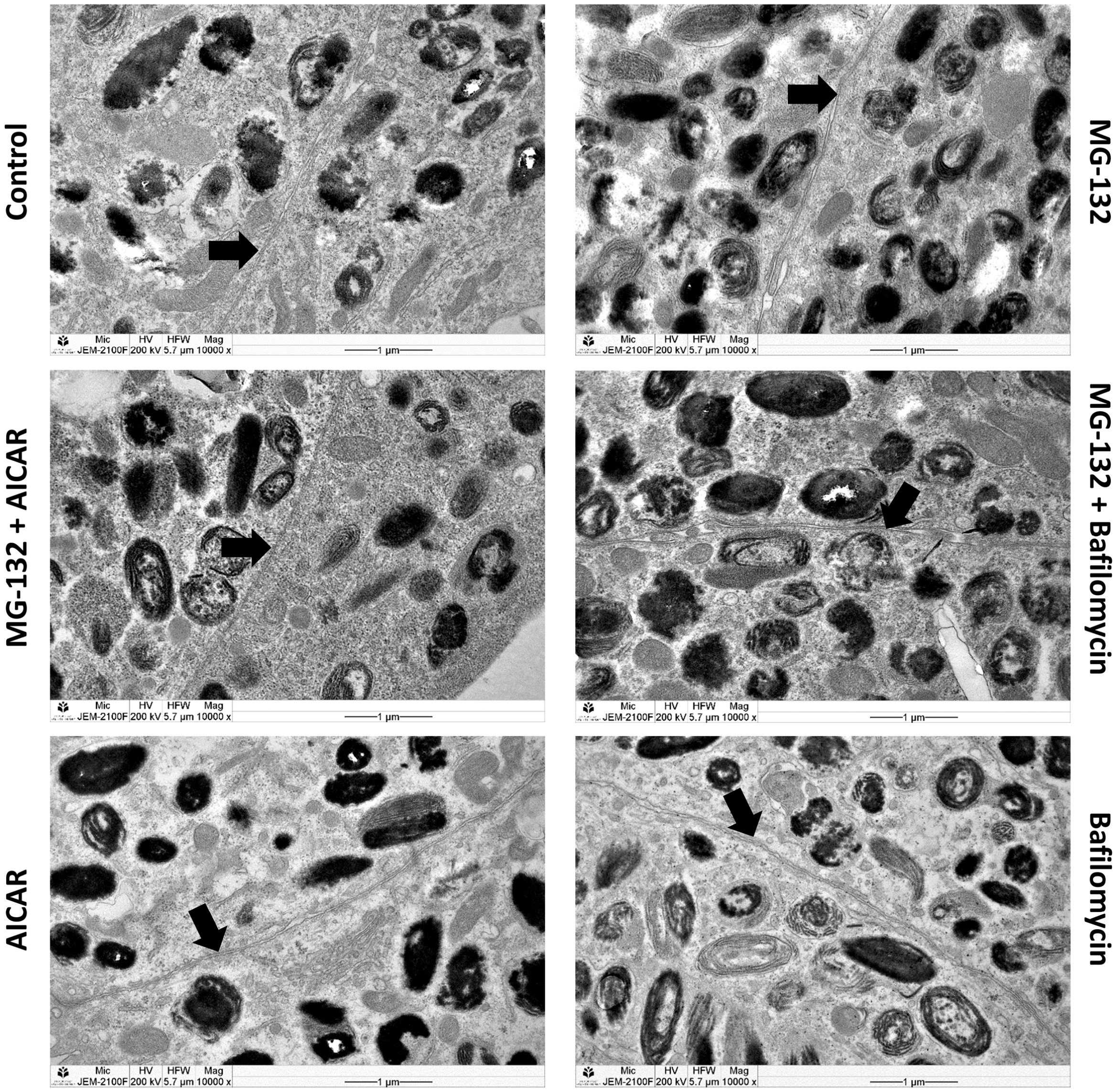

Figure S8: Representative transmission electron micrographs of hESC-RPE cells show cell junctions after exposures to MG-132 (1 µM), AICAR (2 mM) or/and bafilomycin A1 (50 nM) for 24 h. Control cells were exposed to culture medium. Black arrow indicates cell junction.

experiment type	passage	adherent culture period (d)	entire culture period (d)
RT-PCR1	p29+1	112	244
RT-PCR2	p29+2	83	249
RT-PCR3	p31+1	83	235
RT-PCR4	p28+1	136	193
**RT-PCR**	average culture period	104	230
	**duration**	**83-112**	**235-244**

experiment type	passage	adherent culture period (d)	entire culture period (d)
EM1	p29+1	112	244
EM2	p25+ 1	36	158

EM3	p26+2	194	309
EM4	p73+2	166	404
**EM**	average culture period	127	279
	**duration**	**36-194 days**	**158-404 days**

**experiment type**	**passage**	**adherent culture period (d)**	**entire culture period (d)**
WB1	p29+1	112	244
WB2	p29+2	83	249
WB3	p31+1	83	235
WB5	p26+2	194	309
WB6	p28+3	51	380
WB7	p37+3	51	294
WB8	p56+3	129	383
**WB**	average culture period	100	299
	**duration**	**51-194 days**	**244-383 days**

**experiment type**	**passage**	**adherent culture period (d)**	**entire culture period (d)**
IF1	p29+1	112	244
IF2	p29+2	83	249
IF3	p31+1	83	235
IF4	p25+ 1	36	338
IF5	p30+3	77	356
IF6	p54+3	77	197
IF7	p69+3	119	319
IF	average culture period	84	277
	**duration**	**36-119**	**197-356**

**experiment type**	**passage**	**adherent culture period (d)**	**entire culture period (d)**
Live-dead 1	p77_3+3	92	292
Live-dead 1	p22_4+3	71	266
**Live-dead**	average culture period	83	277
	duration	**36-112**	**197-356 days**

**experiment type**	**passage**	**adherent culture period (d)**	**entire culture period (d)**
pigment analysis	p29+1	112	244
pigment analysis	p31+1	83	235
**pigment analysis**	average culture period	98	240
	duration	**83-112**	**235-244 days**
